# Self-Calibration of an Industrial Robot Using a Novel Affordable 3D Measuring Device

**DOI:** 10.3390/s18103380

**Published:** 2018-10-10

**Authors:** Martin Gaudreault, Ahmed Joubair, Ilian Bonev

**Affiliations:** 1 SRI International, Menlo Park, CA 94025, USA; 2 École de Technologie Supérieure (ÉTS), University of Quebec, Montreal, QC H3C 1K3, Canada; ahmed.joubair.1@ens.etsmtl.ca

**Keywords:** precision, robot calibration, robot accuracy, autonomous calibration, closed-loop calibration, self-calibration

## Abstract

This work shows the feasibility of calibrating an industrial robot arm through an automated procedure using a new, low-cost, wireless measuring device mounted on the robot’s flange. The device consists of three digital indicators that are fixed orthogonally to each other on an aluminum support. Each indicator has a measuring accuracy of 3 µm. The measuring instrument uses a kinematic coupling platform which allows for the definition of an accurate and repeatable tool center point (TCP). The idea behind the calibration method is for the robot to bring automatically this TCP to three precisely-known positions (the centers of three precision balls fixed with respect to the robot’s base) and with different orientations of the robot’s end-effector. The self-calibration method was tested on a small six-axis industrial robot, the ABB IRB 120 (Vasteras, Sweden). The robot was modeled by including all its geometrical parameters and the compliance of its joints. The parameters of the model were identified using linear regression with the least-square method. Finally, the performance of the calibration was validated with a laser tracker. This validation showed that the mean and the maximum absolute position errors were reduced from 2.628 mm and 6.282 mm to 0.208 mm and 0.482 mm, respectively.

## 1. Introduction

In the past two decades, metrology equipment and methods for industrial robot arm calibration [1] have progressed tremendously, fueled by an ever-increasing demand for higher accuracy. Most manufacturers no longer want to teach robot poses manually and rely solely on the high repeatability of industrial robots. This approach is inflexible and time-consuming. 

One alternative to reduce the costs associated with this method is to use offline programming software to plan the robot movements. However, because industrial robots are precise but not accurate, this method often results in poor accuracy when the program is transferred to the real robot and requires numerous touchups. Therefore, a calibration procedure is required to increase the robot’s accuracy. Unfortunately, most calibration methods involve expensive measuring devices such as laser trackers. However, this problem can be solved by designing new calibration instruments and methods that, hopefully, provides similar results after calibration at a low cost. 

The ideal robot calibration method should be fully-automated, executable on-site, quick to set up and perform and, of course, highly effective. At the same time, the measuring instruments used for robot calibration should not only be accurate (volumetric accuracy better than 0.1 mm), but also easy to use and affordable.

Current robot calibration methods can be classified into two main categories: open-loop methods and closed-loop methods [2]. Open-loop methods make use of external metrology equipment to measure the partial or full pose of the robot’s end-effector when identifying the robot’s parameters. Over time, researchers have used different metrology systems such as acoustic sensors [3], mechanical coordinate-measuring machines (CMM)s [4], theodolites [5], laser trackers [6,7,8], optical CMMs [9], combinations of the latter two [10], and ballbars [11] for open-loop calibration. These instruments allow for good calibration performances but have significant drawbacks. They require training and are more expensive than devices used for closed-loop calibration. Finally, the measurements can be time-consuming, unless fully-automated, which is not always possible [12].

Closed-loop calibration, also known as self-calibration or autonomous calibration, can be defined as the automated process of identifying the robot’s parameters by using its internal sensors only [13] and possibly a sensor attached to the robot’s end-effector. Researchers have used various devices mounted on a robot’s flange such as ballbars [14], touch probes [15], optical sensors [16], and cable transducers [17] to use as internal sensors. However, the efficiency of such methods has never been excellent.

In this paper, a novel self-calibration method is presented using a new 3D measuring sensor attached to the robot’s flange. The method is almost as effective as the standard calibration procedure involving a laser tracker, but is more affordable. The measuring principle is similar to that proposed in [18]. However, instead of manually constraining the robot’s tool center point (TCP) to a mechanical coupling (e.g., a ball in a socket), the proposed measuring device is used to automatically drive the robot’s TCP to coincide with the center of a datum sphere. This seemingly minor difference is in fact a significant improvement since no frequent manual back-and-forth jogging or force control capability is required and the method can be fully-automated. Furthermore, three datum spheres instead of one are used for measurements, and they form a permanent world reference frame. Thus, the robot’s accuracy is improved with respect to this physical reference frame, not with respect to some imaginary frame as usually done.

On the one hand, instruments similar to the proposed position measuring device already exist, namely ROSY [19] by Teconsult GmbH, Bayreuth, Germany (based on two cameras) and Laser LAB by Wiest AG, Neusäß, Germany (based on five laser sensors). However, not only is the accuracy of these two devices relatively poor (as low as 0.1 mm) but they are used as error measurement devices rather than replacements of a position’s physical constraint. This means that the robot’s TCP is never exactly at the center of the datum sphere, but as far away as 10 mm. While this offset is taken into account in the calibration, it is not measured with high accuracy.

On the other hand, far more accurate devices based on the same principle have been used for calibrating machine tools. Some researchers have proposed a device called the R-Test [20], which uses three analog linear probes and has an uncertainty of 1.7 μm for a measurement range of less than 0.5 mm. A very similar device has also been proposed in [21], but using four probes. Later, these researchers expanded upon their “chase-the-ball” concept by calibrating a five-axis machine [22]. Finally, the company IBS Precision Engineering (Stuttgart, Germany) offers the Trinity contact-free probe, which can measure the offset of the center of a special datum sphere (expensive and fragile) within a range of up to 3.5 mm (still too small for robotics) and with an accuracy of less than 0.001 mm. Unfortunately, all these devices are relatively expensive (the Trinity costs about $15,000), because they need to be built with great accuracy and calibrated before use. They are an excellent measuring solution for machine tools, but inadequate for industrial robots.

**Figure 1:**
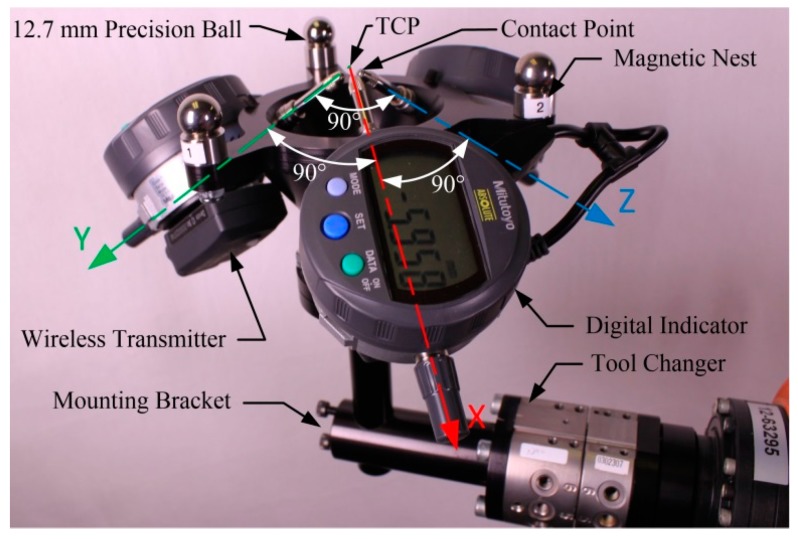
The proposed 3D measuring device.

In contrast, our device (Figure 1) is based on three off-the-shelf digital indicators which have an accuracy of 3 μm. Such devices have already been used for measuring repeatability, i.e., for measuring offsets, albeit very small ones [23]. However, our device is not used to measure offsets with high accuracy, but only to iteratively guide the robot’s TCP towards the center of a datum sphere until all indicators are nearly zeroed. For that reason, it does need tight manufacturing tolerances. However, it requires an affordable means of defining the TCP (the location at which all indicators are zeroed). Therefore, the main novelty of our device and approach is the design and use of a special calibrator plate for defining this TCP.

The proposed measuring device and its accessories (ball plate, kinematic coupling platform) are affordable (cost about $5000) and inexpensive to repair (in the event of a collision), but can be used to position the robot’s TCP onto the center of a datum sphere with an offset of less than the repeatability of the robot (in our study, 0.010 mm). This means that if the coordinates of sufficiently many datum spheres are measured on a CMM, our measurement scheme can be as accurate as when using a laser tracker. Of course, in practice, dozens of such spheres cannot be used, so an important objective is to find a reasonably low number of datum spheres. This keeps the performance of this approach comparable to the performance of robot calibration using a laser tracker.

This device was first described in [24], where it was used to calibrate a small industrial robot. However, the results for the robot’s position accuracy after calibration were rather poor when validated in the robot’s whole workspace. In this paper, there have been significant improvements compared to our previous work. Firstly, the post-calibration results are greatly improved due to the use of a comprehensive mathematical model for the robot. This mathematical model is more complete with the addition of a parameter for consecutive parallel axes. Using this parameter in combination with the level 3 non-kinematic parameters on joints 2–6 yields much better calibration results in the entire workspace of the robot. In other words, it is demonstrated that the measuring device, when used in combination with an appropriate mathematical model and an optimal set of robot configurations, can provide calibration results in the complete workspace of the robot that are more than five times better than the results that were presented in our previous paper. Secondly, a novel methodology was used to validate the performance of each set of parameters found, which is to do multiple identifications (i.e., more than 2000 identifications) of the robot’s parameters. Usually, the authors present the results of their calibration with only one identification of the parameters, which might have been the best results achieved after multiple optimizations, or just plain luck. Besides, most authors present validation results in only a few poses (typically less than 100). This new method to characterize the calibration performance of a measuring instrument gives much more credibility to the results as it shows the impact of the number of configurations selected and what is the range of post-calibration accuracy that can really be achieved with such a device. When a device like the TriCal is used in industry, it is not possible to validate the performance of the calibration with a laser tracker due to budget constraints. Therefore, the engineer must rely on the probabilities that the calibration will be successful. Our new method provides insight into how to evaluate the probabilities of a successful calibration based on the number of measurements. 

This paper is structured as follows. First, the proposed device and reference artifacts, the measuring procedure and uncertainty estimation, and the method and the theory used for identifying the robot’s parameters are described in Section 2. Section 3 presents the experimental results. Finally, the discussion and conclusion are presented in Section 4.

## 2. Materials and Methods

### 2.1. TriCal and Its Accessories

**Figure 2:**
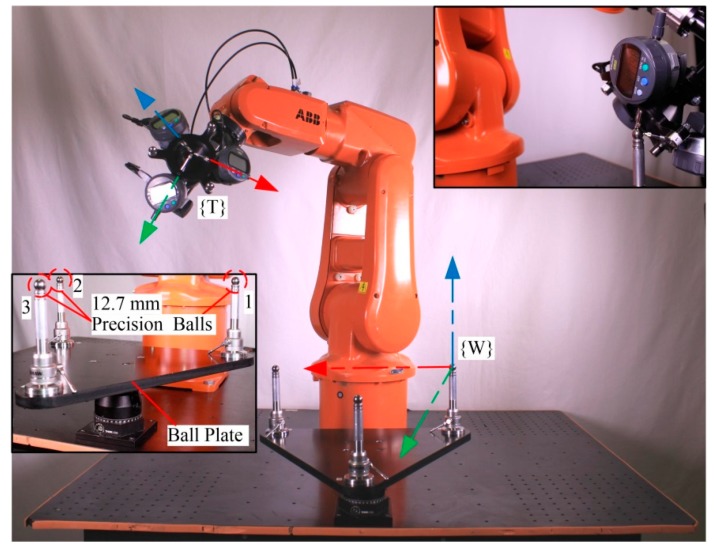
Experimental setup 1.

TriCal, the novel device used in our work (Figure 1), is mounted on the flange of a robot and measures the relative position of stationary 12.7 mm (0.5 in) precision balls (Figure 2) with the help of three digital indicators. It is, however, important to understand that the accuracy of TriCal is not uniform. The device is highly accurate only when the center of the ball is in the vicinity of the TCP, where all digital indicators show no more than a few micrometers. In other words, TriCal is a device used only to bring the robot’s TCP to a known position with respect to the robot’s base.

TriCal (Figure 1) consists of three Mitutoyo ID-C112XB indicators (Kanagawa Prefecture, Japan). Each indicator has an accuracy of 0.003 mm and a measuring range of 12.7 mm. The indicators are supported by an aluminum conical bracket and are orthonormal to each other. Finally, three magnetic nests for 12.7 mm balls (from Hubbs Machine and Manufacturing) are fixed at the extremities of the conical bracket.

**Figure 3:**
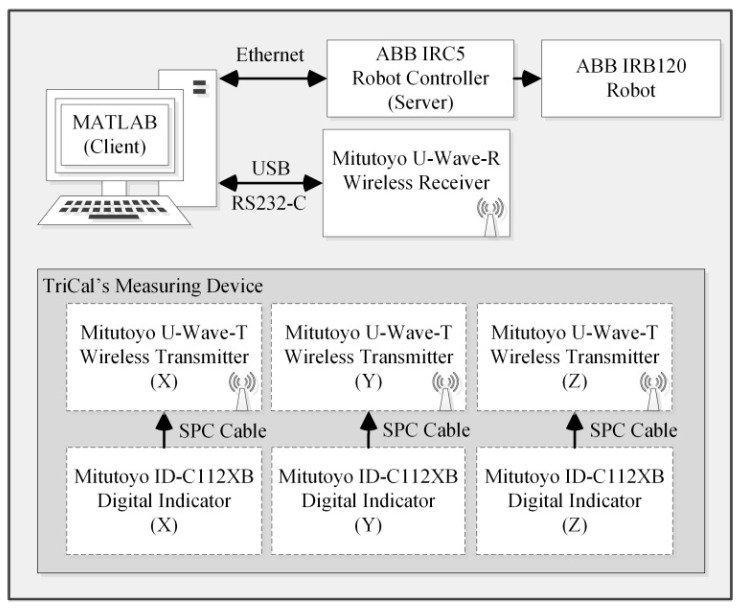
Communication setup.

Each digital indicator is connected through a statistical process control (SPC) cable to a Mitutoyo U-WAVE-T wireless transmitter which transmits the measurements to a Mitutoyo U-WAVE-R wireless receiver. The receiver, which is connected through a universal serial bus (USB) cable to a personal computer (PC), stores the data from the transmitters as soon as a measurement changes. This outcome is achieved by setting the transmission parameters to “Event Driven Mode.” In order to retrieve the measurements stored in the receiver’s memory, an American Standard Code for Information Interchange (ASCII) string is sent from MATLAB 2014a. The information acquired in MATLAB is then sent to the robot’s controller via a local area network. The communication setup between the robot, the PC and TriCal is presented in Figure 3.

**Figure 4:**
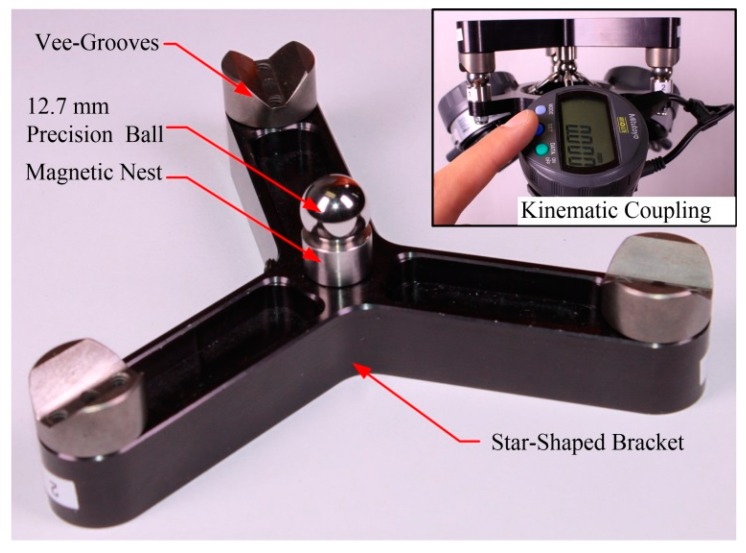
Kinematic coupling platform.

As already mentioned, TriCal is used to bring a virtual TCP to a specific position. Therefore, a crucial step is the ability to precisely define this TCP with respect to the TriCal’s body. This is achieved through the use of a special kinematic coupling platform (Figure 4), which is essentially a star-shaped aluminum fixture holding a magnetic nest in its center (from Hubbs Machine and Manufacturing, Cedar Hill, AL, USA) and three vee-blocks (from Bal-tec, Los Angeles, CA, USA) at its extremities. The purpose of the kinematic platform is to locate a 12.7 mm precision ball at the TCP of the device described in the previous section, in a highly repeatable manner. Once the kinematic platform is positioned over the measuring device, as shown in Figure 4, all three digital indicators are zeroed (with their “set” buttons). It has been demonstrated that this TCP position is highly repeatable by coupling and decoupling the kinematic platform from the TriCal’s body multiple times and reading the measurement values on the three digital indicators. Those values were still 0.000 mm every time the kinematic coupling platform was constrained between the vee-grooves. The same configuration, out of the three possible mating configurations, must be used for the repeatability to be 0.000 mm. Therefore, the location of the TCP can be measured precisely by a CMM by making sure that the same configuration is used.

Finally, an arbitrary number of datum spheres is required for gathering measurements with the TriCal. In this paper, for simplicity, only three datum spheres are used, because their relative positions can be measured promptly using a ballbar from Renishaw (Gloucestershire, UK). Furthermore, the placement of those three datum spheres is not optimized with respect to the robot’s base. The problem of choosing the optimal number of datum spheres and their optimal placement will be studied in the future.

The ball plate (Figure 2) used in this study is composed of an aluminum triangular platform with three magnetic nests for 12.7 mm balls mounted on risers from Renishaw. The plate is mounted on an articulating platform from Thorlabs (Newton, NJ, USA), which allows the operator to vary the orientation of the platform. The nests are placed approximately 300 mm apart so that the exact distance between them can be measured with a telescoping ballbar from Renishaw. Furthermore, nests and balls are utilized instead of tooling balls, so that they are used to easily validate our work with a ION laser tracker from FARO (Lake Mary, FL, USA), by replacing the balls with 12.7 mm spherically mounted reflectors.

### 2.2. Measurement Procedure

The measurement procedure can be divided into two main operations: semi-automated steps and a fully-automated centering procedure. Note that several semi-automated steps are needed only the first time the device is used on a particular robot cell. The whole calibration process was executed on an ABB IRB 120 robot equipped with the new measuring device, as depicted in Figure 2. This particular setup will be referred to as setup 1 (measurements for identification).

The measuring device is used to gather measurements, but before it can be used safely and in an automated fashion, three semi-automated steps should be executed. The first semi-automated step is performed to define a reference position on each of the digital indicators by using the kinematic platform. To do so, three 12.7 mm precision balls are positioned on the magnetic nests of the measuring device. Then, the vee-grooves of the kinematic platform are mated to the precision balls. The operator resets each indicator to zero (0.000) when the platform is fully-constrained onto the measuring device. This step takes approximately one minute to complete.

The second semi-automated step is performed to identify the position of the TCP with respect to the wrist of the robot. This procedure requires four robot configurations. To register each of the required joint targets, the operator must jog the robot until the three stems of TriCal are in contact with any one of the three precision balls of the ball plate. Then, the operator can start an automated centering procedure. This procedure is programmed both in MATLAB and in RAPID and is explained later. Its purpose is to move the measuring device until all three digital indicators display “0.000”. Once reached, the current joint target (robot configuration) is saved in the robot’s controller. This process is repeated three times on the same precision ball that was selected for the first measurement. The position of the TCP with respect to the wrist can be found by minimizing the Cartesian errors at the end-effector. This can be accomplished by using the forward kinematic equations and an approximate TCP position, as is usually done by industrial robot manufacturers to identify the TCP location. The second semi-automated step takes approximately 15 min to complete.

Once the TCP has been found, it is used to identify the positions of each of the three precision balls on the ball plate in the robot’s internal coordinate system. The measuring device must be brought to each of the three balls by jogging. Then, on each ball, the automated centering procedure is executed. When all three indicators show “0.000”, the robot pose is saved. The step takes approximately 20 min.

As soon as the semi-automated steps are completed, the measurements can be collected automatically on each of the three 12.7 mm precision balls through an automated centering procedure (i.e., once the robot’s TCP coincides with the center of one of the three balls, we take the angle readings of the six joints). The algorithm is shown in Algorithm 1. For the experiment, the maximum error, *ε*, was set at 0.010 mm. Ideally, this value should be 0. However, if the value is below the robot’s position repeatability, the time to measure one configuration will double in some cases. At *ε* = 0.010 mm, each robot configuration takes approximately 20 s to be measured.

**Algorithm 1** Automated Centering Procedure1: **Procedure** AutoCenter($q_{d}$)  # $q_{d}$ is the desired joints configuration to measure 2: MAX_EPSILON ← 0.010   # 0.010 mm = position repeatability 3: Move the robot to $q_{d}$    # from MATLAB to Controller 4: Wait 1 s                # to ensure effective communication 5: Send data request             # from MATLAB to U-WAVE-R 6: $r_{x}\leftarrow$ Measurement of indicator along the tool X  # from U-WAVE-R to MATLAB 7: $r_{y}\leftarrow$ Measurement of indicator along the tool Y  # from U-WAVE-R to MATLAB 8: $r_{z}\leftarrow$ Measurement of indicator along the tool Z  # from U-WAVE-R to MATLAB 9: $r\leftarrow \left[r_{x},r_{y},r_{z}\right]$ 10: **while**(norm(r)) >= MAX_EPSILON **do** 11:  Move the robot’s TCP by vector r         # from MATLAB to Controller 12:  Wait 1 s                     # to ensure effective communication 13:  Send data request                # from MATLAB to U-WAVE-R 14:  $r_{x}\leftarrow$ Measurement of indicator along the tool X  # from U-WAVE-R to MATLAB 15:  $r_{y}\leftarrow$ Measurement of indicator along the tool Y  # from U-WAVE-R to MATLAB 16:  $r_{z}\leftarrow$ Measurement of indicator along the tool Z  # from U-WAVE-R to MATLAB 17: $r\leftarrow \left[r_{x},r_{y},r_{z}\right]$ 18: **return**
$q_{a}\leftarrow$ Actual joints configuration        # from Controller to MATLAB

The only measurements that can be collected with Setup 1 are the positions of the centers of the three balls with respect to {W}. Let $p_{T}^{W}$ be the position vector of {T} with respect to {W}, and let *d_ij_* be the distance measured between the centers of balls *i* and *j* (*i* = 1, 2, 3; *j* = 1, 2, 3; *i* ≠ *j*). The three position vectors that can be measured, which represent the centers of balls 1, 2, and 3, are:

(1) $$p_{T,1}^{W}=[0,0,0],$$

(2) $$p_{T,2}^{W}=[d_{12},0,0],$$

(3) $$p_{T,3}^{W}=[p_{x},p_{y},0],$$

where:

(4) $$p_{x}=\frac{d_{12}^{2}+d_{13}^{2}-d_{23}^{2}}{2d_{12}},$$

and:

(5) $$p_{y}=\sqrt{d_{13}{}^{2}-p_{x}{}^{2}}.$$

The distances between each pair of magnetic nests were measured with a Renishaw QC-20W ballbar. The rationale for using a ballbar instead of a traditional CMM is that it is more accurate but also much cheaper. 

A total of 360 randomly-generated robot configurations (120 robot configurations per precision ball) were subsequently measured with this experimental setup. 

The measurement uncertainties of this new calibration method are caused by the inaccuracy of the digital indicators, the mechanical tolerances on each component, the experimental conditions and the measuring procedure. Specifically, Table 1 and Table 2 show the uncertainty estimation associated with each source of errors for the measuring device and the measuring procedure, respectively. The TriCal is used as a constraining device, thus some of the sources of errors are negligible or can be reduced significantly by adjusting the parameters and conditions of the calibration process. For instance, the maximum angular deviation (0.122°) of the digital indicators might seem important. However, when considering that the robot constrains the measuring device within 0.010 mm with respect to the center of the 12.7 mm precision ball, the projected source of error is orders of magnitude smaller than the other sources of errors. Also, if the calibration can be performed in a temperature-controlled environment, the sources of error related to thermal expansion can be neglected. Furthermore, if more time can be spent on the calibration, the automated centering tolerance *ε* can be lowered to 0, thus reducing the sources of errors. In these conditions, and assuming the fact that those errors represent a worst case scenario (i.e., all those sources of errors add up), the measuring device’s absolute accuracy is approximately 9 µm, which is less than the robot`s repeatability (10 µm). It is important to note that the uncertainty associated with the hysteresis and the friction of very small end-effector displacements was not quantified within the automated centering error. The negative effects of this type of uncertainty can be diminished by moving the robot with larger movements. More precisely, the robot end-effector can be moved away significantly from the target precision ball, with the condition that it should maintain contact with it. Then, using the measurements on the three indicators, another single movement attempt can be made to move the end-effector directly on the target within the desired tolerance. This procedure can be repeated until the robot is finally at the desired location. However, the calibration would take more time to perform. The combined maximum error would be less than 27 µm if the sources of errors of the measuring procedure are added to those of the measuring device.

### 2.3. Calibration Model and Identification Method

An accurate identification of the robot calibration model’s parameters is crucial to improve the absolute position accuracy. The measurements set used for identification should also be optimal for the identification method that is employed. To that end, an observability optimization was performed on a large pool of robot configurations to select the optimal configurations for the identification process. Then, the method of least squares was used to identify the robot parameters using the optimized robot configurations.

**Figure 5:**
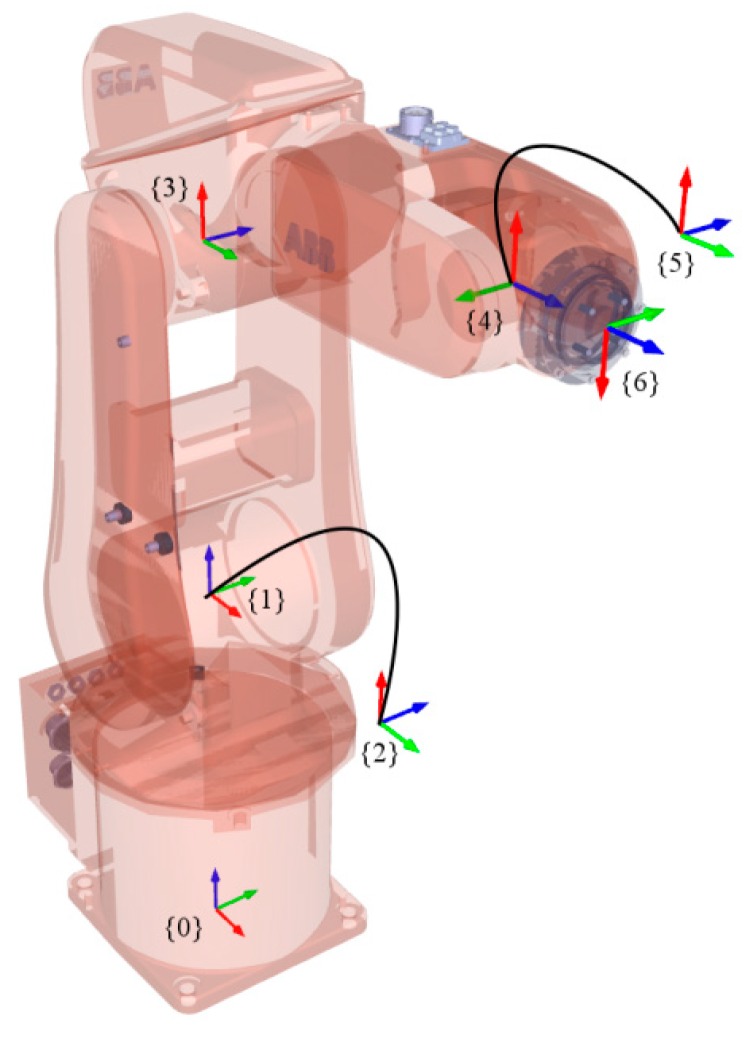
Reference frames associated with the robot arm.

The robot was modeled using the Denavit-Hartenberg (D-H) parameters as per Craig’s convention [25]. Furthermore, an additional parameter was added to consider consecutive parallel axes [26]. Figure 5 shows all the link frames. The base frame is denoted by {0}. The robot’s nominal parameters are shown in Table 3.

The homogeneous matrix linking each successive pair of frames of the robot is represented as:

(6) $$T_{i}^{i-1}=R_{X}\left(\alpha_{i-1}\right)D_{X}\left(a_{i-1}\right)R_{Y}(\beta_{i-1})R_{Z}\left(\theta_{i}\right)D_{Z}\left(d_{i}\right),$$

where $\alpha_{i-1},\;a_{i-1},\;\theta_{i},\;\mathrm{and}\;d_{i}$ are the D-H parameters, $s\theta_{i}=\sin\theta_{i},$ $c\theta_{i}=\cos\theta_{i}$, **R***_Q_* is the homogeneous rotation matrix around axis *Q*, **D***_Q_* is the homogeneous translation matrix along *Q*, and $T_{i}^{j}$ is the homogeneous matrix representing the pose of frame {*i*} with respect to frame {*j*}. The rotation parameter, $\beta_{i-1}$, addresses the problem of the proportionality of the model [27]. To obtain the homogeneous matrices of the base frame {0} with respect to the world frame {W}, and of the tool frame {T} with respect to the flange frame {6}, the following equation was used:

(7) $$T(\chi )=D_{X}\left(x\right)D_{Y}\left(y\right)D_{Z}\left(z\right)R_{Z}\left(\alpha \right)R_{Y}(\beta )R_{X}\left(\gamma \right),$$

and the parameters are presented in Table 4.

The calibration model includes a total of 31 parameter errors: 26 kinematic parameters and 5 non-kinematic parameters, as seen in Table 5 and Table 6. The parameter errors associated with link 1 are not considered, because they are dependent on the base parameters. Also, axes 2 and 3 are parallel, so only one of either *δd*_2_ or *δd*_3_ should be included in the calibration model. Therefore *δd*_2_ was arbitrarily chosen for removal. The tool parameters are also not included for identification because the position of the tool is measured with a 3-axis CMM with respect to the robot’s last axis frame. Note that these parameters do not need to be measured frequently, as long as TriCal is manipulated with care. The tool orientation parameters cannot be incorporated into the model because the measuring instrument provides only three-dimensional position measurements.

The compliance in each gearbox is modeled as a linear torsional spring, as presented in [6]. The compliance in the gearbox of the first joint is not included because no torque is applied to this joint when the robot is not moving, as the joint axis is vertical. The torque on each of the other five joints is calculated with the iterative Newton-Euler algorithm [25].

First, let us define the vector of all constant parameters of the robot model to be:

(8) $$\rho ={\left[\begin{array}{cccccccc} \alpha & a & d & \theta_{\mathrm{offs}} & c & \chi_{B} & \chi_{T} & \beta_{2} \end{array}\right]}^{T},$$

where $\chi_{B}$ and $\chi_{T}$ are the vectors of the parameters of the base and the tool, respectively. Then, by using forward kinematics, the pose of {T} with respect to {W} can be expressed as a function of the constant parameters (ρ) and the variable parameters (q,τ):

(9) $$T_{T}^{W}(\rho ,q,\tau )=T_{0}^{W}T_{6}^{0}T_{T}^{6}.$$

Now, the position vector of the homogeneous matrix can be defined to be:

(10) $$x=\left[\begin{array}{c} T_{T}^{W}{}_{1,4}\\ T_{T}^{W}{}_{2,4}\\ T_{T}^{W}{}_{3,4} \end{array}\right]=\left[\begin{array}{c} x_{T}^{W}\\ y_{T}^{W}\\ z_{T}^{W} \end{array}\right].$$

By assuming that the parameter errors are small, the difference between the position measurements of one configuration (i.e., $x_{mes},y_{mes},z_{mes})$ and the position obtained by calculating the forward kinematics (i.e., *x*, *y*, *z*) of the calibrated model is:

(11) $$\left[\begin{array}{c} x_{mes}-x\\ y_{mes}-y\\ z_{mes}-z \end{array}\right]=\left[\begin{array}{c} \Delta x\\ \Delta y\\ \Delta z \end{array}\right]=\Delta x=\frac{\partial x}{\partial \rho }\Delta \rho =J\Delta \rho ,$$

where J is the Jacobian matrix of x. The concatenation of all the *n* measurements that are used for identification is expressed as:

(12) $$\left[\begin{array}{c} \Delta x_{1}\\ \Delta x_{2}\\ \vdots \\ \Delta x_{n} \end{array}\right]=\left[\begin{array}{c} J_{1}(\rho ,q_{1},\tau_{1})\\ J_{2}(\rho ,q_{2},\tau_{2})\\ \vdots \\ J_{n}(\rho ,q_{n},\tau_{n}) \end{array}\right]\Delta \rho ,$$

which can also be written as:

(13) ΔX=JΔρ

To find the variation of the parameters Δρ, the identification Jacobian must be inverted. However, since the identification Jacobian is not square, the following equation is used:

(14) $$\Delta \rho ={\left(J^{T}J\right)}^{-1}J^{T}\Delta X,$$

which is equivalent to:

(15) $$\Delta \rho =J^{+}\Delta X,$$

where $J^{+}$ is the expression of the Moore–Penrose inverse. 

Next, an observability assessment was performed in order to obtain the best sets for identifying the robot’s parameters. The observability index *O*_1_ [28] was selected for optimization as it had been previously demonstrated to give better results when the robot model incorporates non-kinematic parameters [29]. The mathematical formula for this observability index is:

(16) $$O_{1}=\frac{{\left(\sigma_{1}\sigma_{2}\ldots \sigma_{m}\right)}^{\frac{1}{m}}}{\sqrt{n}},$$

where *n* is the number of configurations in the set, *m* is the number of parameters of the model, and $\sigma_{i}$ are the singular values of the identification Jacobian matrix. To optimize the observability index of a set of *n* robot configurations, the DETMAX algorithm was used [30] on a large initial pool of *N* robot configurations. When the DETMAX algorithm finishes, it outputs a set of *n* robot configurations with an optimal observability index. The robot’s parameters are then identified using this optimal set of robot configurations with the least squares optimization method, as presented in Equations (11)–(15).

## 3. Results

In this section, a dispersion analysis of the identification procedure using multiple optimized sets of robot configurations of various sizes is introduced. Then, the performance of the calibration using a set of 75 optimized robot configurations for identification is presented. Next, the performance of this new method is compared to an earlier calibration method using the same calibration device. Finally, the results of this new calibration method are compared with the results of other works performed on the same robot.

### 3.1. Calibration Dispersion Analysis

Several parameter identifications were performed to assess the dispersion of the calibration results when using different set sizes. More precisely, a total of 360 robot configurations were initially measured using setup 1 (i.e., using TriCal as a constraining device on the three precision balls of the ball plate). Then, multiple sets containing between 20 and 100 robot configurations were formed using those 360 configurations. Thirty identifications were performed on each set size (i.e., in each identification, the number of robot configurations is the same, but not the configurations themselves). Furthermore, for each set, the observability index was optimized using the initial pool size of 360 randomly generated robot configurations. 

**Figure 6:**
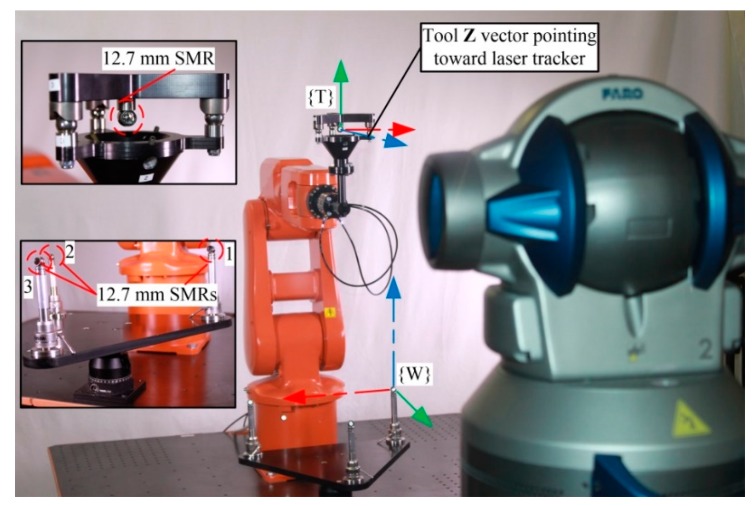
Experimental setup 2.

The experimental setup on which the robot configurations were measured for validation purposes is shown in Figure 6, and it is referred to as setup 2. This setup still makes use of TriCal, but with a few modifications in order to be able to measure the position of the center ball with a laser tracker. First, the digital indicators were removed from the conical bracket. Then, the 12.7 mm precision ball of the calibrator was replaced by a 12.7 mm spherically mounted retroreflector (SMR), and the calibrator was locked onto the measuring device using a kinematic coupling and rubber bands (not shown). Using balls and an SMR of identical diameters makes it possible to measure exactly the same TCP position with the laser tracker. Finally, the 12.7 mm precision balls of the ball plate were replaced by 12.7 mm SMRs to measure the TCP with respect to exactly the same {W}. The laser tracker was placed at approximately two meters in front of the SMR 3. According to the manufacturer’s specifications, this laser tracker has a point-to-point typical accuracy of 32 µm when using a 2.3 m horizontal scale bar measurement at a measurement distance of five meters from the laser tracker. It is also important to note that the laser tracker was used for validation purposes only, and not to acquire measurements to identify the robot parameters. 

**Figure 7:**
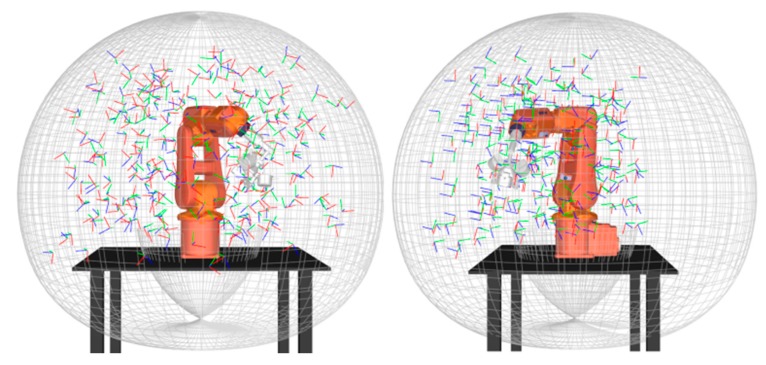
Robot tool poses measured with experimental setup 2.

The absolute position errors after calibration were measured with a laser tracker at 506 randomly-generated robot configurations in the complete robot workspace (Figure 7). The only constraint on these configurations is that the SMR is visible to the laser tracker.

**Figure 8:**
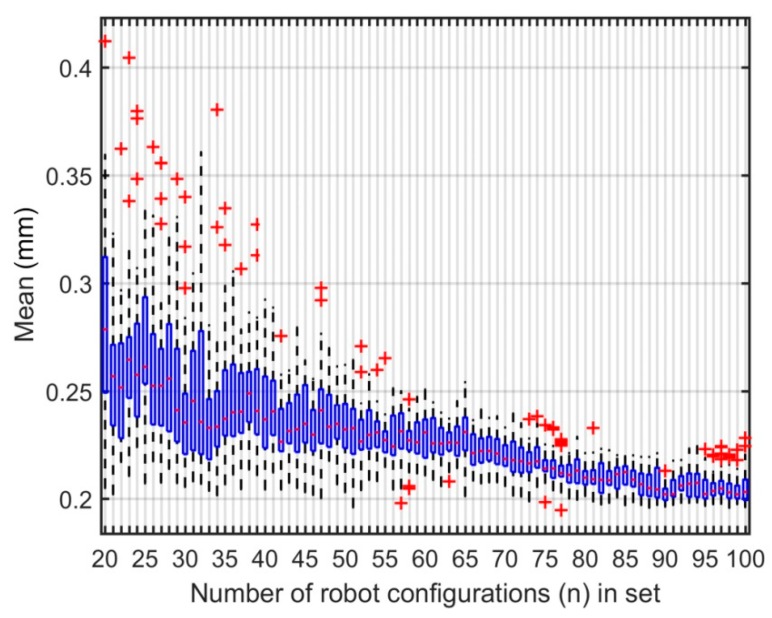
Box and whisker plot, mean of absolute position errors after calibration.

**Figure 9:**
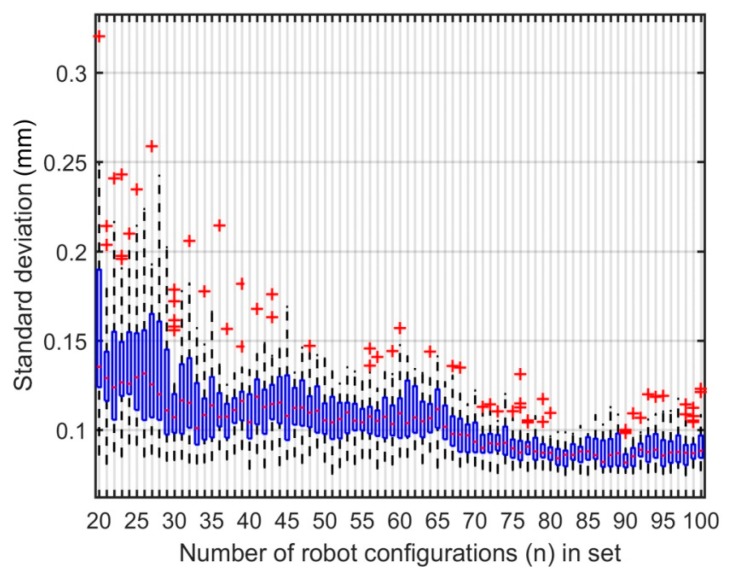
Box and whisker plot, standard deviation of absolute position errors after calibration.

The multiple means and standard deviations of the absolute position errors after calibration are displayed as quartiles on a box and whisker plot as shown in Figure 8 and Figure 9, respectively. On those plots, the red crosses are outliers. It can be noted that the performance of the calibration varies significantly for different sets of robot configurations of the same size even when the observability index is optimized. This is especially true for smaller sets (i.e., sets composed of fewer robot configurations used in the identification). Also, the data shows a clear trend. The sets that contain more robot configuration for identification will generally give better calibration results. Consequently, at 75 configurations, the calibration results are similar to those of larger sets. Table 7 shows the descriptive statistics of the identified parameters for 30 sets of 75 robot configurations. According to these statistics, the identified parameters are stable with low standard deviations. This outcome correlates with the low dispersion in the calibration performances.

### 3.2. Absolute Position Errors

**Figure 10:**
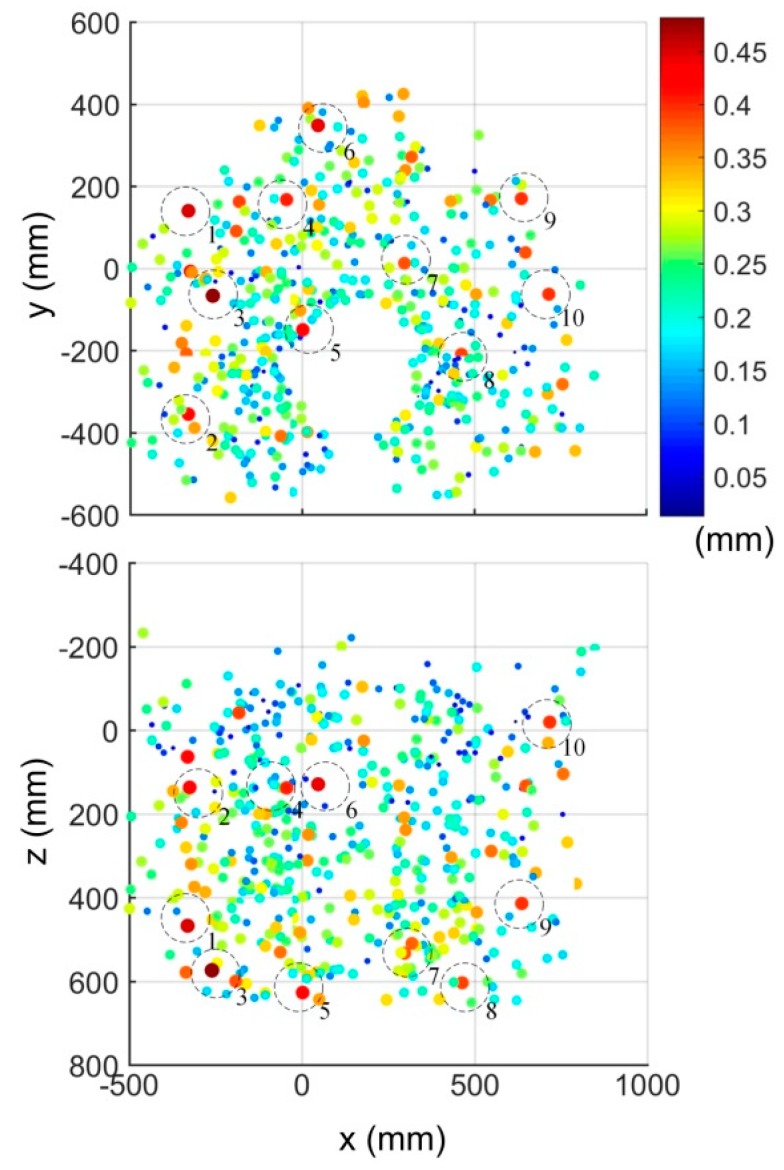
Scatter plot of absolute position errors after calibration.

**Figure 11:**
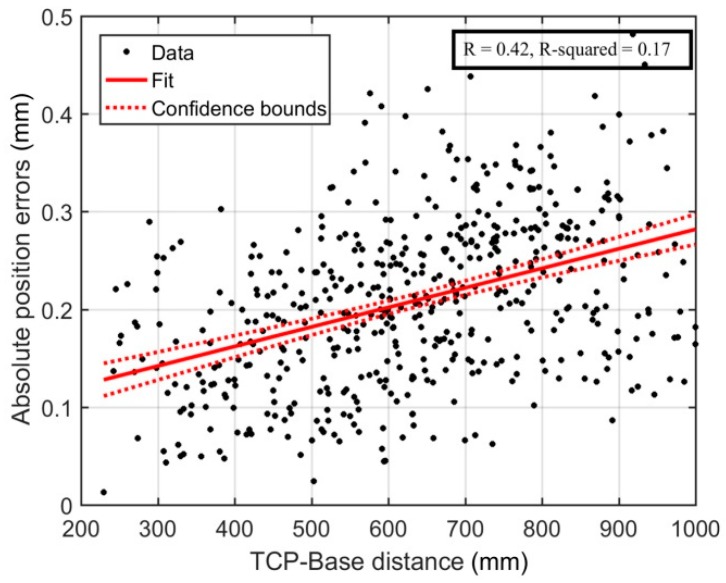
Linear regression-Distance between the TCP and the origin of the base frame.

**Figure 12:**
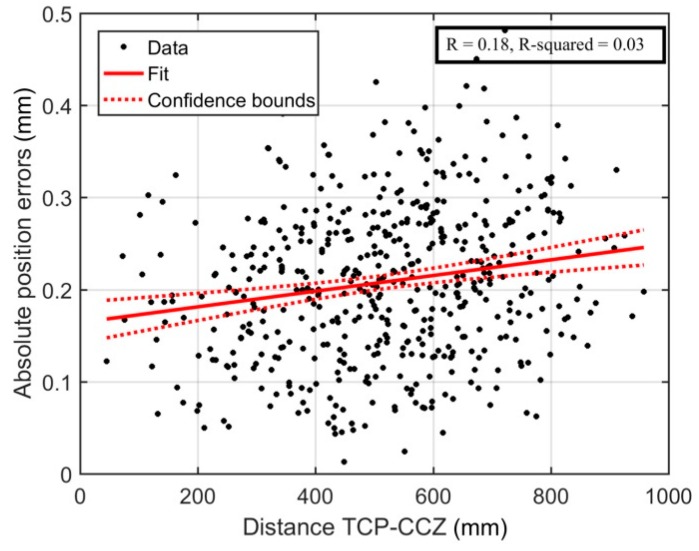
Linear regression-Distance between the TCP and the center of the calibration zone.

**Figure 13:**
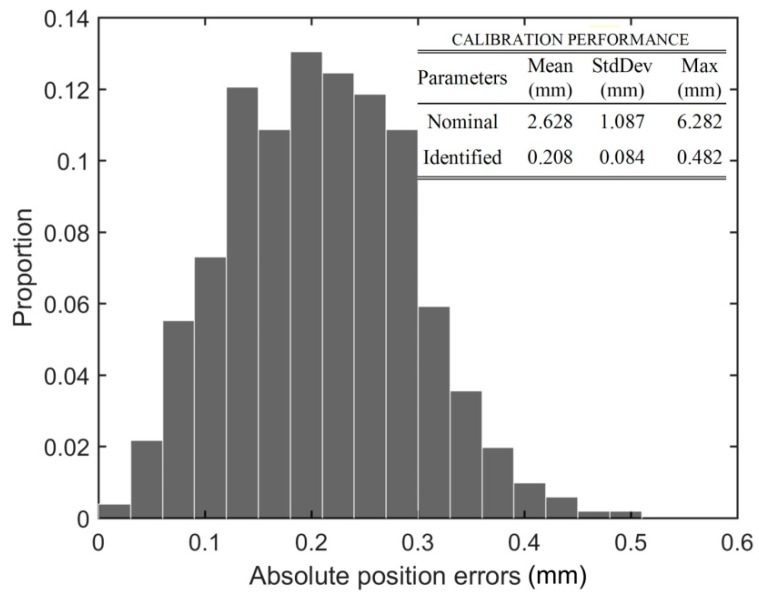
Histogram of absolute position errors at the TCP after calibration.

The absolute position errors were plotted on the 2D scatter plot shown in Figure 10. The locations of the 10 biggest errors are encircled. Furthermore, two linear regression analyses were performed, one for the absolute position errors with respect to {0}, and the other with respect to the center of the calibration zone (CCZ). The CCZ is located at the center of gravity of the triangle formed by the three measurement positions. The regression analyses are shown in Figure 11 and Figure 12, respectively. Finally, Figure 13 shows the distribution of absolute position errors after calibration as well as the descriptive statistics of the errors before and after calibration.

For all these analyses, the parameters of the model were identified with one optimized set of 75 robot configurations using setup 1 (Figure 2) and validated by setup 2 (Figure 6) on the same 506 configurations as previously described (Figure 7). 

Figure 10 shows that the largest errors are primarily located at the extremities of the robot’s workspace. The regression analysis presented in Figure 11 confirms the weak linear relationship between the distance from the TCP to the base of the robot and the absolute precision errors. 

Naturally, errors are expected to be higher when the arm is fully extended. However, Figure 12 shows no linear relationship between the distance from the TCP to the CCZ and the absolute precision errors. In other words, this result shows that this calibration method provides good performance throughout the whole workspace even though the measurements are collected in a restricted volume in front of the robot.

### 3.3. Comparison of TriCal with Other Calibration Methods

Table 8 shows a comparison between different measurement methods that were performed on the same IRB120 robot. Our method identifies the parameters of the robot model through 30 optimized sets of 75 robot configurations measured with setup 1. With the TriCal, the performances were validated with the same 506 robot configurations in setup 2.

In the case of C-Track, an optical CMM from Creaform (Lévis, QC, Canada), the TCP was a half-sphere with a retroreflective target, while in the case of the laser tracker, an SMR was used (as in the validation of setup 2) [9]. For the identification of the robot’s parameters with the laser tracker and the optical CMM, the same least squares method and observability index optimization algorithm were used. Once again, in all three cases, the results were validated with a laser tracker. The data show that the performance of TriCal with three base-mounted spheres is slightly lower than the performance of the laser tracker or the optical CMM. However, the cost of the TriCal is also significantly lower. This comparison might seem unfair since the cost of building the prototype is compared against the acquisition cost of those measuring devices. However, the TriCal can easily be custom built.

In reality, all those calibration methods require expert knowledge and a significant amount of time. The time spent on each calibration method depends on many variables. Those variables are the number of configurations required for the identification of the robot’s parameters, the number of configurations required for the validation of its accuracy after calibration, the setup time, which can vary depending on the work cell constraints, the experience of the user with the measuring technology, the speed of the robot, etc. Thus, a process time comparison between the different measuring technologies is subjective and inappropriate.

## 4. Discussion

In this paper a novel low-cost, three-dimensional automated measuring device (TriCal) and a robot calibration procedure were presented. TriCal was used to calibrate a six-axis serial industrial robot. It was shown that the TriCal device is nearly as good as a laser tracker for calibrating a small industrial robot. Namely, it was possible to reduce the absolute position errors to 0.482 mm (maximum), as verified in more than 500 random robot configurations. 

The cost of the new 3D measuring device is significantly lower than any other used for robot calibration in industry. For example, a laser tracker typically costs more than $100,000, while TriCal’s prototype costs approximately $5000. Moreover, an annual calibration of a laser tracker, let alone a repair, costs thousands of dollars whereas an accident involving TriCal would incur repair costs of no more than several hundred dollars.

In addition to its low acquisition costs, TriCal is less sensitive to variations in atmospheric conditions than a laser tracker. In an industrial environment where temperature, humidity and vibrations cannot be controlled, the TriCal is a safe alternative for field calibration. The TriCal device can even be purposed for measuring position repeatability for bigger robots.

The TriCal is arguably among the best measuring tools for performance evaluation and calibration of industrial robots, especially for small and medium enterprises that cannot afford an expensive measuring device for the sole purpose of robot calibration. We are in the process of commercializing this tool.

One very important study that remains to be done, however, is on the optimal number and placement of the datum spheres. Ideally, these datum spheres must be fixed on the same plate on which the robot is fixed, and their positions should be measured on a CMM, with respect to the actual base of the robot. These datum spheres should remain part of the robot cell, even after calibration. They can be used for automated periodical validation of both the accuracy and the repeatability of the industrial robot.

## Figures and Tables

**Table 1:** Sources of errors of the measuring device.

Sources of Errors	Uncertainty
Accuracy of each digital indicator ID-C112XB	3.00 µm
Tolerance on diameter of measuring balls	2.50 µm
Tolerance on diameter of contact point spheres of indicators	NA
Maximum angular deviation of digital indicators (machining)	0.122°
Projected angular deviation considering *ε* = 0.010 mm	0.02 µm
Combined maximum error	~9 µm

**Table 2:** Sources of errors related to the measuring procedure.

Sources of Errors	Uncertainty
Automated centering error	10.00 µm
Accuracy of ballbar	3.70 µm
Accuracy of the coordinate-measuring machine (CMM) (MT Mitutoyo Bright Strato 7106)	2.7 um (95% confidence interval)
Approx. thermal expansion of ball plate (Δ0.2°C)	2.07 µm
Approx. thermal expansion of measuring device (Δ0.2°C)	1.38 µm
Approx. thermal expansion of ball support stems (Δ0.2°C)	0.36 µm
Combined errors (using assumption that errors add up)	20.21 µm

**Table 3:** ABB IRB120 nominal parameters.

i	α_i−1_ (°)	a_i−1_ (mm)	d_i_ (mm)	θ_i_ (°)	β_i−1_ (°)
1	0	0	290	*q*_1_	-
2	−90	0	0	*q*_2_ − 90	-
3	0	270	0	*q*_3_	0
4	−90	70	302	*q*_4_	-
5	90	0	0	*q*_5_	-
6	−90	0	72	*q*_6_ + 180	-

**Table 4:** Tool and base nominal parameters.

Frame	x (mm)	y (mm)	z (mm)	α (°)	β (°)	γ (°)
Tool${}^{\chi_{T}}$	$x_{T}^{6}$	$y_{T}^{6}$	$z_{T}^{6}$	$\alpha_{T}^{6}$	$\beta_{T}^{6}$	$\gamma_{T}^{6}$
World${}^{\chi_{B}}$	$x_{0}^{W}$	$y_{0}^{W}$	$x_{0}^{W}$	$\alpha_{0}^{W}$	$\beta_{0}^{W}$	$\gamma_{0}^{W}$

**Table 5:** Robot calibration parameters.

i	α_i−1_(°)	a_i−1_ (mm)	d_i_ (mm)	θ_i_ (°)	β_i−1_(°)
1	*α*_0_ + *δα*_0_	*a*_0_ + *δa*_0_	*d*_1_ + *δd*_1_	*q*_1_ + *θ_offs_*_,1_ + *δθ_offs_*_,1_ + *c*_1_*τ*_1_	-
2	*α*_1_ + *δα*_1_	*a*_1_ + *δa*_1_	*d*_2_ + *δd*_2_	*q*_2_ + *θ_offs_*_,2_ + *δθ_offs_*_,2_ + *c*_2_*τ*_2_	-
3	*α*_2_ + *δα*_2_	*a*_2_ + *δa*_2_	*d*_3_ + *δd*_3_	*q*_3_ + *θ_offs_*_,3_ + *δθ_offs_*_,3_ + *c*_3_*τ*_3_	*β*_2_ + *δβ*_2_
4	*α*_3_ + *δα*_3_	*a*_3_ + *δa*_3_	*d*_4_ + *δd*_4_	*q*_4_ + *θ_offs_*_,4_ + *δθ_offs_*_,4_ + *c*_4_*τ*_4_	-
5	*α*_4_ + *δα*_4_	*a*_4_ + *δa*_4_	*d*_5_ + *δd*_5_	*q*_5_ + *θ_offs_*_,5_ + *δθ_offs_*_,5_ + *c*_5_*τ*_5_	-
6	*α*_5_ + *δα*_5_	*a*_5_ + *δa*_5_	*d*_6_ + *δd*_6_	*q*_6_ + *θ_offs_*_,6_ + *δθ_offs_*_,6_ + *c*_6_*τ*_6_	-

**Table 6:** Tool and base calibration parameters.

Frame	x (mm)	y (mm)	z (mm)	α (°)	β (°)	γ (°)
Tool	xT6+δxT6	yT6+δyT6	zT6+δzT6	αT6+δαT6	βT6+δyT6	γT6+δγT6
World	$x_{0}^{W}+\delta x_{0}^{W}$	$y_{0}^{W}+\delta y_{0}^{W}$	$z_{0}^{W}+\delta z_{0}^{W}$	$\alpha_{0}^{W}+\delta \alpha_{0}^{W}$	$\beta_{0}^{W}+\delta \beta_{0}^{W}$	$\gamma_{0}^{W}+\delta \gamma_{0}^{W}$

**Table 7:** Identified parameters from 30 optimized sets of 75 configurations.

Parameters	Nominal	Mean	StdDev	Min	Max
*α*_1_ (°)	−90.000	−90.034	0.001	−90.035	−90.032
*α*_2_ (°)	0.000	−0.045	0.001	−0.046	−0.043
*α*_3_ (°)	−90.000	−90.011	0.001	−90.013	−90.009
*α*_4_ (°)	90.000	89.998	0.000	89.997	89.999
*α*_5_ (°)	−90.000	−89.990	0.001	−89.991	−89.990
*a*_1_ (mm)	0.000	0.013	0.008	−0.010	0.032
*a*_2_ (mm)	270.000	270.146	0.004	270.137	270.153
*a*_3_ (mm)	70.000	70.120	0.007	70.106	70.131
*a*_4_ (mm)	0.000	−0.075	0.001	−0.078	−0.073
*a*_5_ (mm)	0.000	−0.012	0.003	−0.019	−0.007
*d*_3_ (mm)	0.000	−0.094	0.004	−0.104	−0.086
*d*_4_ (mm)	302.000	302.296	0.011	302.276	302.315
*d*_5_ (mm)	0.000	0.019	0.002	0.015	0.023
*d*_6_ (mm)	72.000	72.163	0.003	72.155	72.171
*θ_offs,_*_2_	−90.000	−89.910	0.004	−89.917	−89.899
*θ_offs,_*_3_	0.000	0.097	0.002	0.091	0.101
*θ_offs,_*_4_	0.000	−0.013	0.001	−0.014	−0.011
*θ_offs,_*_5_	0.000	0.062	0.001	0.060	0.063
*θ_offs,_*_6_	180.000	180.259	0.001	180.258	180.260
*c*_2_ (°/Nm × 10^−3^)	0.000	−2.060	0.099	−2.245	−1.924
*c*_3_ (°/Nm × 10^−3^)	0.000	−5.522	0.149	−5.809	−5.274
*c*_4_ (°/Nm × 10^−3^)	0.000	−33.738	0.611	−34.955	−32.153
*c*_5_ (°/Nm × 10^−3^)	0.000	−30.448	0.527	−31.875	−29.426
*c*_6_ (°/Nm × 10^−3^)	0.000	−44.555	3.047	−51.723	−39.429
*β*_2_ (°)	0.000	0.039	0.001	0.038	0.040
$x_{0}^{W}$ (mm)	160.211	158.865	0.006	158.867	158.873
$y_{0}^{W}$ (mm)	−255.727	−253.835	0.013	−253.861	−253.809
$z_{0}^{W}$ (mm)	−220.548	−219.170	0.017	−219.190	−219.128
$\alpha_{0}^{W}$ (°)	91.603	91.677	0.001	91.675	91.679
$\beta_{0}^{W}$ (°)	0.496	0.148	0.003	0.142	0.155
$\gamma_{0}^{W}$ (°)	−1.384	−1.380	0.000	−1.381	−1.379

**Table 8:** Comparison of TriCal with other calibration methods.

Instrument	Median (mm)	StdDev (mm)	Max (mm)	Approximate Cost ($)
TriCal	0.208	0.084	0.482	5000
Laser tracker	0.146	0.065	0.437	>100,000
Optical CMM	0.176	0.081	0.492	>50,000

## Supplementary Materials

All procedures described in this paper, including the validation, are detailed in a six-minute video available at https://youtu.be/Tvj-IwQmVBw.
